# 

**Published:** 1905-08-15

**Authors:** 


### ANNOUNCEMENTS.

The following is a list of the number of graduates from each of the colleges in the United States for the session of 1904 and 1905.

| Name of College. | Number Graduates. |
|---|---|
| Baltimore College of Dental Surgery | 79 |
| University of Maryland | 76 |
| Baltimore Medical College | 31 |
| Pennsylvania College of Dental Surgery | 98 |
| Philadelphia Dental College | 113 |
| University of Pennsylvania | 165 |
| Medico-Chirurgical College | 15 |
| New York College of Dental Surgery | 104 |
| New York College of Dental and Oral Surgery | 54 |
| University of Buffalo | 74 |
| Harvard University | 30 |
| Tufts College | 26 |
| George Washington University | 23 |
| Georgetown University | 5 |
| Howard University | 9 |
| Medical College of Virginia | 6 |
| University College of Medicine, Richmond | 18 |
| Southern Dental College | 40 |
| Atlanta Dental College | 81 |
| University of Tennessee | 37 |
| Vanderbilt University | 36 |
| Mahary Dental College | 13 |
| Birmingham Dental College | 11 |
| New Orleans College of Dental Surgery | 31 |
| Ohio College of Dental Surgery | 77 |
| Cincinnati College of Dental Surgery | 22 |
| Ohio Medical University | 56 |
| Western Reserve University | 30 |
| Pittsburg Dental College | 61 |
| University of Michigan | 34 |
| Detroit College of Medicine | 15 |
| Indiana Dental College | 71 |
| Louisville | 105 |
| Chicago Dental College | 168 |
| Northwestern University | 207 |
| University of Illinois | 69 |
| Milwaukee Medical College | 28 |
| Milwaukee College of Physicians and Surgery | 6 |
| University of Minnesota | 48 |
| University of Iowa | 16 |
| Keokuk Dental College | 18 |
| St. Louis Dental College | 67 |
| Washington University | 59 |

| | |
|---|---|
| Barnes Dental College | 7 |
| Kansas City Dental College | 48 |
| Western Dental College | 57 |
| Omaha Dental College | 33 |
| Lincoln Dental College | 6 |
| Colorado Dental College | 17 |
| North Pacific Dental College | 41 |
| University of California | 40 |
| College of Physicians and Surgeons | 36 |
| University of Southern California | 20 |
| Total | 2627 |

---

NORTHERN INDIANA DENTAL SOCIETY.—The next meeting of this society will be held September 19th and 20th, at Logansport.

---

AMERICAN SOCIETY OF ORTHODONTISTS.—The next meeting of this society will be held September 28th to 30th, at Chicago.

---

DR. MICHAEL C. SHEHAN, a graduate of the University of Michigan, and practising in Detroit, died June 10th.

---

DAMAGE SUITS.—A New York City dentist has been sued for $10,000 on the claim that he did not properly treat a broken jaw.—A man in Buffalo has brought suit against the Dental College claiming that his son was inoculated with dental instruments which were not properly sterilized.—An advertising dentist of Los Angeles, Cal., was assessed $2,400 damages by the judge of the superior court because in extracting some teeth from a ladies mouth be allowed one of the roots to fall into the trachea, where it lodged for several months to the great distress of the patient.

OHIO BOARD OF EXAMINERS.—The Governor of Ohio has renominated the members of the Ohio Board, Drs. H. C. Brown, J. K. Douglas, L. L. Barber, Henry Barnes and Stanley Smith. This board has worked harmoniously and done good service, and we are pleased that the Governor approves of their work.

—✦—

ANOTHER CHANGE OF DEANS.—Dr. Harry P. Carlton has resigned as Dean of the Dental Department of the University of California, and Dr. James G. Sharp has been elected his successor. Harry is a capital fellow and a very efficient man and the cause of education loses a valuable supporter.

—✦—

DR. HERMAN PRINZ, Editor of *Dental Era*, was married July 11th to Miss Lillie Koop, of St. Louis. A St. Louis paper gives an extended write up of the romance of the affair, and also details some pleasantries which his friends in the building where he offices contributed to the occasion.

—✦—

THE MICHIGAN STATE DENTAL ASSOCIATION held one of its best meetings at Detroit, July 10th to 12th. The meeting was largely attended, and the program was good and instructive. The social features were a great success and the Detroit dentists deserve great praise for the generous hospitality extended their guests. The next meeting will be held at Saginaw. Dr. J. J. Green, of Ionia, was elected President; Dr. C. H. Warboys, of Albion, Trustee. The other officers were re-elected.

